# REPRODUCIBILITY OF BRAIN AGE SALIENCIES ACROSS DEEP NEURAL NETWORK ARCHITECTURES

**DOI:** 10.1093/geroni/igad104.3572

**Published:** 2023-12-21

**Authors:** Nicholas Kim, Phoebe Imms, Nikhil Chaudhari, Nahian Chowdhury, Anar Amgalan, Paul Bogdan, Andrei Irimia

**Affiliations:** University of Southern California, Los Angeles, California, United States; University of Southern California, Los Angeles, California, United States; University of Southern California, Los Angeles, California, United States; University of Southern California, Los Angeles, California, United States; University of Southern California, Los Angeles, California, United States; University of Southern California, Los Angeles, California, United States; University of Southern California, Los Angeles, California, United States

## Abstract

The brain’s biological age (BA) reflects structural changes related to neuroanatomic senescence. BA is estimated from magnetic resonance images (MRIs) using convolutional neural networks (CNNs), which provide interpretability through saliency maps. By conveying the importance of brain regions to BA estimation, these maps reveal sex differences in brain-aging. We compared two CNN architectures to assess the reproducibility of such differences. Both regression CNNs had T1-weighted MRIs as inputs; outputs were estimated BAs. One CNN was trained on males and females separately, whereas the other was trained on both sexes. The dataset used consisted of T1-weighted MRIs from 5,851 cognitively normal individuals (3,142 females) aged 22 to 95 years, sampled from the Alzheimer’s Disease Neuroimaging Initiative (N = 510), Human Connectome Project Aging (N = 508) and Young Adult (N = 1,112), and UK Biobank (N = 3,721). For both models, compared to the average saliency for a null distribution, males’ BA estimation relied significantly (p < 0.05) more on the right lateral temporal lobe and superior frontal gyrus. Females’ BA estimation depended significantly (p <; 0.05) more on the right posterior and bilateral occipital regions, and medial parietal lobe. Simulated alterations in brain morphometry indicated saliencies correctly revealed regions with aging-related dynamics, which confirm prior findings on sex differences in brain-aging. Variations in CNN models’ saliencies did not affect overall anatomic patterns, suggesting that CNNs can capture brain-aging patterns robustly despite architecture differences. Novel statistical models for formal comparison of CNN saliencies should be developed to accommodate their nonlinear nature.

